# Analyzing the Interaction between Clinical, Neurophysiological and Psychological Outcomes Underlying Chronic Plantar Heel Pain: A Network Analysis Study

**DOI:** 10.3390/ijerph191610301

**Published:** 2022-08-18

**Authors:** Marta Ríos-León, Juan Antonio Valera-Calero, Ricardo Ortega-Santiago, Umut Varol, César Fernández-de-las-Peñas, Gustavo Plaza-Manzano

**Affiliations:** 1 Hospital Nacional de Parapléjicos, SESCAM, 45004 Toledo, Spain; 2 Department of Physiotherapy, Faculty of Health, Universidad Camilo José Cela, Villanueva de la Cañada, 28692 Madrid, Spain; 3 VALTRADOFI Research Group, Department of Physiotherapy, Faculty of Health, Universidad Camilo José Cela, Villanueva de la Cañada, 28692 Madrid, Spain; 4 Department of Physical Therapy, Occupational Therapy, Rehabilitation and Physical Medicine, Universidad Rey Juan Carlos, 28922 Alcorcón, Spain; 5 Cátedra Institucional en Docencia, Clínica e Investigación en Fisioterapia: Terapia Manual, Punción Seca y Ejercicio Terapéutico, Universidad Rey Juan Carlos, 28922 Alcorcón, Spain; 6 Department of Radiology, Rehabilitation and Physiotherapy, Universidad Complutense de Madrid, 28040 Madrid, Spain; 7 Grupo InPhysio, Instituto de Investigación Sanitaria del Hospital Clínico San Carlos (IdISSC), 28040 Madrid, Spain

**Keywords:** plantar heel pain, risk factors, foot function, depression, trigger points

## Abstract

Plantar heel pain (PHP) is one of the most common foot pain conditions in adults. Several biological and psychological factors could be involved in chronic PHP in a complex matrix. However, reciprocal interactions between these factors are unknown. The aim of the present study was to use network analysis to quantify potential multivariate relationships between pain-related, function, clinical, mechanosensitivity, psychological, and health-related variables in individuals with PHP. Demographic (age, gender), pain-related (pain intensity), function, clinical (myofascial trigger points [TrPs]), mechanosensitivity (pressure pain thresholds), psychological (Beck Depression Inventory), and health-related variables (EQ-5D-5L) were collected in 81 PHP patients. Network connectivity analysis was conducted to quantify the adjusted correlations between the modeled variables and to assess their centrality indices. The connectivity network showed local associations between pain-related variables, foot function, and mechanosensitivity. Additionally, associations between quality of life, depression, and pain-related variables were found, while TrPs was associated with quality of life and mechanosensitivity. The node with the highest strength centrality was the worst pain intensity, while mechanosensitivity and worst pain intensity showed the highest closeness and betweenness centrality. This is the first study to apply network modeling to understand the connections between pain-related, function, clinical, mechanosensitivity, psychological, and health-related variables in PHP. The role of pain severity and mechanosensitivity is highlighted and supported by the network. Thus, this study reveals potential factors that could be the target in the management of PHP, promoting a comprehensive and effective therapeutic approach.

## 1. Introduction

Plantar heel pain (PHP) is one of the most common causes of foot pain in adults [1]. It is characterized by throbbing medial plantar heel pain, especially during the first step in the morning or after a prolonged period of inactivity [2,3]. Its prevalence ranges from 4% to 9.6% [4] and it is estimated that it occurs in approximately two million Americans each year [5,6,7], resulting in over one million physician visits annually in the United States of America (USA) [5,6]. In addition, up to 20% of PHP patients continue with symptoms longer than one year [8], leading to a significant negative impact on foot-specific and general health-related quality of life due to functional limitations in a broad range of activities and physical tasks [9]. Therefore, this condition can be highly disabling in almost 8% of the population [4], and the associated annual economic burden accounts for USD 284 million [10]. Despite the impact and prevalence of PHP, its etiology remains controversial making it difficult to determine effective treatments and preventive programs [11].

Previous studies have identified several factors involved in a complex matrix behind the potential etiopathogenesis of PHP [8,12,13,14,15,16,17]. Among these factors, foot-level findings (e.g., limited ankle joint dorsiflexion) [8], presence of active trigger points (TrPs) [12], reduced muscle strength and endurance [8], and psychological factors [16,17] could be involved in PHP in a complex matrix. In addition, emerging evidence also supports the presence of altered central nociceptive pain processing in people with PHP [13,14] related to pressure pain hyperalgesia [13,15]. Supporting these associations, some previous studies have reported different interactions between clinical, psychological, and neurophysiological variables in people with PHP [12,13,15,18]. However, these studies used Spearman’s rank-order correlation, Pearson’s Product-Moment Correlations, or linear regressions to determine the associations between the outcomes [12,13,15,18]. It should be noted that Spearman’s rank-order correlation or Pearson’s Product-Moment Correlation ignore the potential for pairwise associations to arise from third-variable effects, whereas linear regressions ignore the possibility of bidirectional relationships between the variables [19].

Network analysis techniques allow a better understanding of complex relationships addressing the mentioned limitations (despite the first step being based on partial correlations) [20]. Network analysis can provide a method to identify the most important variables in the associated complex network. Thus, this analysis could be used to potentially design better therapeutic strategies for improving the management of PHP [21]. From a network perspective, PHP can be viewed as a complex condition sustained by mutual interactions between clinical, physiological, and psychological systems. Previous studies have used network analysis for a better understanding related to the complexity of chronic pain syndromes [22,23].

However, no study has applied network analysis in PHP research. As the current PHP framework considers the reciprocal interactions between biological and psychological factors, this analysis could add precision to research on PHP and develop more targeted management procedures. Therefore, the aims of this study were: (1) to apply a network connectivity analysis including pain-related, function, clinical, mechanosensitivity, psychological, and health-related variables in individuals with PHP; and (2) to illustrate the potential of a network analysis for understanding underlying features of PHP and improving options for developing more targeted and effective treatment strategies. Based on the available literature [12,13,14,15,16,17,18,22,23,24,25], we hypothesized that pain intensity, foot function, number of trigger points, mechanosensitivity, depression, and quality of life will show multiple associations in individuals with PHP.

## 2. Materials and Methods

### 2.1. Study Design

An observational cross-sectional study following the Strengthening the Reporting of Observational Studies in Epidemiology (STROBE) guidelines [26] was conducted. The study design was approved by the Local Ethics Committee (URJC 051220160022020). All participants signed an informed consent before their inclusion in the study.

### 2.2. Participants

Individuals with a primary report of plantar heel pain attending a physical therapy clinic in Madrid (Spain) between January 2020 and April 2022 were screened for eligibility criteria. Inclusion criteria were: (1) adults aged 18 years or older; (2) clinical diagnosis of plantar heel pain according to the clinical practice guidelines from the Orthopaedic Section of the American Physical Therapy Association (i.e., insidious onset of sharp pain on the plantar heel surface on weight bearing after a period of non–weight bearing, pain increasing in the morning with the first step after waking up, and tenderness with palpation of the proximal insertion of the plantar fascia) [7]; and (3) unilateral plantar heel pain for more than 3 months.

Exclusion criteria were: (1) previous surgery within the lower extremity; (2) presence of positive neurologic signs related to nerve root compression; (3) other medical condition associated with heel pain (e.g., rheumatoid arthritis, peripheral neuropathy); or (4) treatment for the heel received within the previous 6 weeks.

### 2.3. Patients’ Assessment

#### 2.3.1. Pain Intensity

An 11-point numerical pain rating scale (NPRS), ranging from 0 (no pain) to 10 (maximum pain), was used to determine the pain intensity [27]. Participants rated their pain intensity at first step in the morning, the mean pain intensity during the day after periods of rest, and the worst level of pain experienced the preceding week on the NPRS.

#### 2.3.2. Function

Foot function was assessed with the Foot Function Index (FFI) [28], a valid and reliable questionnaire for several foot disorders such as plantar heel pain [29]. The FFI consists of 23 self-reported items divided into 3 subcategories: pain (9-items), disability (9-items), and activity limitation (5-items). Each item is scored on a scale from 0 (no pain or difficulty) to 10 (worst pain or so difficult it requires help).

Subscale scores range from 0% to 100%, with higher scores indicating lower levels of function and worse foot health-related quality of life [29]. The FFI total score (i.e., mean of the 3 subscale scores) was used in the analysis.

#### 2.3.3. Clinical Presentation

Since the finding of myofascial trigger points (TrPs) was related to symptoms experienced by people with plantar heel pain [30], the total number of TrPs detected in the examination of each subject was also recorded. The presence of TrPs was explored in the internal gastrocnemius, flexor hallucis brevis, adductor hallucis, and quadratus plantae muscles according to international diagnostic criteria (i.e., presence of a sensitive spot into a taut band of a skeletal muscle that elicits referred pain in response to manual compression) [31].

A TrP was considered active if the elicited referred pain reproduced symptoms of the patient, whereas a TrP was considered latent if the elicited referred pain did not reproduce any symptoms considered familiar to the patient [31]. The order of evaluation was randomized between subjects, with a two-minute rest period between muscles.

#### 2.3.4. Mechanosensitivity

Pressure pain threshold (PPT), defined as the minimal amount of pressure where a sensation of pressure changes to pain [32], was used to evaluate local and widespread pressure pain sensitivity. PPTs were bilaterally assessed with an electronic algometer (FPIX^TM^, Wagner Instruments, Greenwich, CT, USA) over different musculoskeletal structures and nerve trunks including the main symptomatic area (calcaneus bone: origin of the plantar fascia), one adjacent area (calcaneus bone: midpoint of calcaneal tuberosity), one segmental-related area (tibialis anterior muscle) and one peripheral nerve (sural nerve).

Pressure was applied at a rate of approximately 1 kg/cm^2^/s on each point [33]. The mean of 3 trials on each point, with a resting period of 30 s for avoiding temporal pain summation [34], was calculated and used for the main analysis. The reliability of PPT assessment over these structures has been found to range from moderate to high [35,36].

#### 2.3.5. Psychological Health

Affective, cognitive, and somatic symptoms of depression were assessed with the Beck Depression Inventory (BDI-II), a 21-item self-report questionnaire [37]. This questionnaire ranked level of depression according to the following ranges of cut-off scores: 0–13 (minimal), 14–19 (mild), 20–28 (moderate), and 29–63 (severe) [37].

#### 2.3.6. Quality of Life

Health-related quality of life was assessed with the paper-based five-level version of EuroQol-5D (EQ-5D-5L) [38,39]. This questionnaire evaluates mobility, self-care, daily activities independency, perceived pain, and anxiety/depression impact domains. Responses range from 1 (absence of problems) to 3 (severe problems). All responses were converted into a single index number, which corresponds to the health state ranging from 0 (health state equivalent to death) to 1 (optimal health) according to standardized values [40].

### 2.4. Network Analysis

All statistical analyses were run in R software v.4.1.1 (RStudio, Boston, MA, USA) for Windows 10. In order to conduct all analyses required, the following three libraries were installed and used: qgraph v.1.6.9 (for network estimation), glasso v.1.11 (for network estimation), and bootnet v.1.4.3 (for stability analyses).

This network was built based on the following 13 variables set as nodes: age, gender, pain intensity after rest, pain intensity during the first step, worst pain intensity, FFI, depression, Euro-Qol-5D, PPTs at tibialis anterior, PPT at sural nerve, PPT at plantar fascia, PPT at calcaneus bone and number of TrPs (latent and active). All nodes were included as continuous except gender (included as categorical). Therefore, since five to ten data points per node are needed for an acceptable sample size according to Hair et al. [41], a minimum of 65 participants would be required.

Edges in the network were represented by lines linking the nodes if associated. Stronger associations were expressed with thicker lines while weaker associations are visualized as thinner lines. Directions of the partial correlations are visualized as red lines for negative correlations and green for positive correlations. In connections involving categorical nodes where sign is not defined, lines were grey [19].

The network structure was determined based on the importance of each node based on centrality indices (i.e., strength, closeness and betweenness) [22]:

- Strength centrality is a blunt measure that takes node’s total level of involvement in the network and not the number of connections with other nodes, being clinically useful to determine which outcomes should be targeted for inducing direct changes in other variables.
- Closeness, which is defined as the inverse sum of the distances of shortest paths of the target node from all other nodes in the network, was interpreted as the expected speed of arrival of something flowing through the network. Therefore, targeting outcomes with high closeness could induce changes to other nodes more quickly than the nodes that are peripheral.
- Betweenness centrality was interpreted as the percentage of shortest paths that must go through the target node. Therefore, a node with a high betweenness centrality would act as an intermediary in the transmission of information or resources between other nodes or even clusters of nodes in the network.

Finally, edge weights and variability of centrality indices were assessed running a bootstrapping with 95% confidence interval (CI) and 1000 iterations. The edge weights bootstrapped CIs were interpreted as accuracy of the estimated weights since only the edges with non-zero weights were preserved. For assessing the variability of the centrality indices (CS-coefficient as a measure of correlation stability), participant-dropping subset bootstrap was utilized. This method reflects the maximum proportion of data that could be dropped to retain >0.7 of the correlation with the original centrality indices [42].

## 3. Results

A sample of 81 participants was screened for participation and analyzed. We had no data loss in this study. Descriptive data of the sample is described in Table 1.

**Figure 1:**
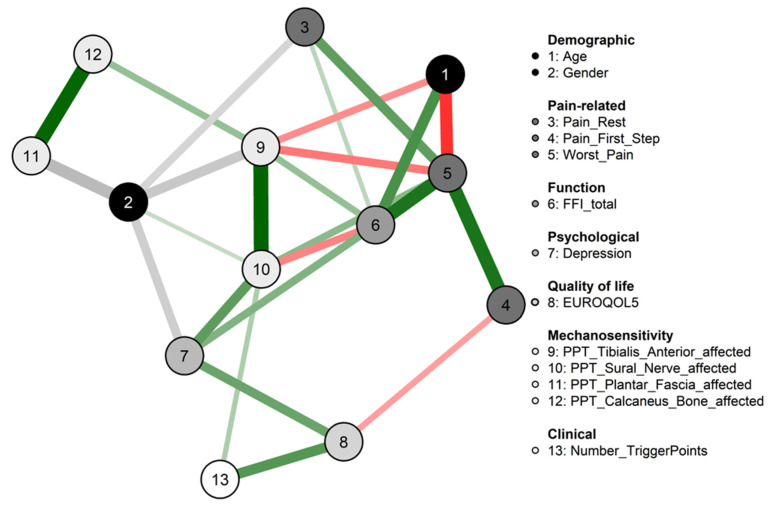
Network analysis of the association between demographic, pain-related, function, psychological, quality of life, and clinical presentation in patients with unilateral chronic heel pain. Edges represent connections between two nodes and are interpreted as the existence of an association between two nodes, adjusted for all other nodes. Each edge in the network represents either positive regularized adjusted associations (green edges) or negative regularized adjusted associations (red edges). The thickness and color saturation of an edge denotes its weight (the strength of the association between two nodes). Abbreviatures: FFI, Foot Function Index; PPT, Pain Pressure Threshold.

The network obtained from this sample is displayed in Figure 1. The strongest associations were found between PPTs locations (i.e., sural nerve with tibialis anterior ρ: 0.51 and calcaneus bone with plantar fascia ρ: 0.50). Regarding the pain-related variables, multiple associations were found. For instance, worst pain intensity was associated with the pain suffered after the first step (ρ: 0.46), foot function (ρ: 0.45), age (ρ: −0.41), and PPT at the tibialis anterior location (ρ: −0.23). Several nodes were linked with foot function, such as PPT at the sural nerve and tibialis anterior locations (ρ: −0.25 and 0.21 respectively), depression (ρ: 0.25), and age (ρ: 0.36). On the other hand, quality of life was associated with pain intensity after the first step (ρ: −0.19) and depression (ρ: 0.30). Finally, the number of TrPs was associated with quality of life (ρ: 0.34) and PPT at the sural nerve location (ρ: 0.16).

**Figure 2:**
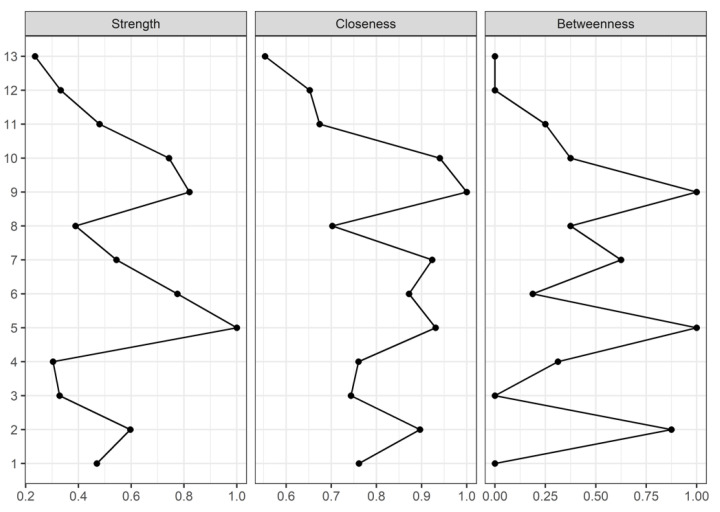
Centrality measures of Strength, Closeness, and Betweenness of each node in the network. Centrality value of 1 indicates maximal importance, and 0 indicates no importance. The nodes’ numbers are consistent with Figure 1.

Centrality indices are shown in Figure 2. The worst pain intensity (node 5) was the node with higher strength centrality, followed by PPT at tibialis anterior (node 9) and foot function (node 10). Regarding the closeness centrality, PPT at tibialis anterior (node 9) and sural nerve locations (node 10), depression (node 7) and worst pain intensity (node 5) were the highlighted nodes. Finally, PPT at the tibialis anterior (node 9), worst pain intensity (node 5), and gender (node 2) were the nodes showing the greatest betweenness centrality.

**Figure 3:**
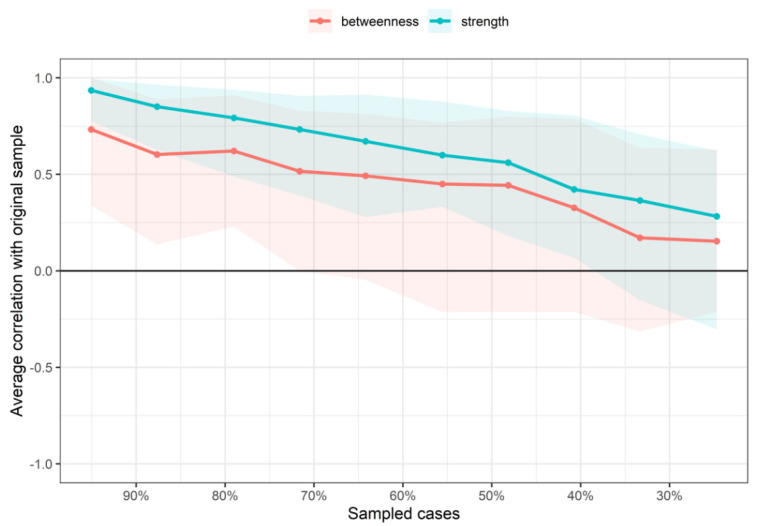
Average correlations between centrality indices of networks sampled with persons dropped and networks built on the entire input dataset. Lines indicate the means and areas indicate the range from the 2.5th quantile to the 97.5th quantile.

The betweenness and closeness measures of the network were unstable at CS_cor=0.7_ = 0.00 and CS_cor=0.7_ = 0.00, respectively. However, the strength centrality measure was found to be relatively stable with CS_cor=0.7_ = 0.05 (Figure 3).

## 4. Discussion

Current understanding supports the presence of several linked biopsychosocial factors underlying features of PHP. This study applied network connectivity analysis to understand the multivariate interaction between pain-related, function, clinical, mechanosensitivity, psychological, and health-related variables in individuals with PHP. Consistent with modern theories on PHP features, the identified network supports a complex model where pain-related, function, clinical, mechanosensitivity, psychological, and health-related variables interact.

The main findings revealed that mechanosensitivity (PPTs), foot function, and age were factors related to pain severity (i.e., worst pain intensity) in individuals with chronic PHP. In addition, mechanosensitivity (PPTs) was also associated with foot function. The presence of pressure pain hypersensitivity and its association with higher pain intensity and limited foot function in individuals with chronic PHP has been reported in previous studies [13,18]. In fact, previous studies reported that 20.8% of pain intensity could be explained by foot function (contribution 2.8%) and calcaneus bone PPTs (contribution 18%) in PHP [18]. According to those results, the current study has shown that the edges with the strongest weight were PPTs (i.e., calcaneus bone with plantar fascia, and sural nerve with tibialis anterior muscle), revealing the importance of pressure pain hyperalgesia in segmental-related areas and symptomatic areas for understanding and management of PHP. These findings support the role of peripheral nociception in the plantar area and the presence of central sensitization related to long-lasting and sustained peripheral noxious input into the central nervous system in individuals with PHP [13,15,18]. Nevertheless, age has also been associated with pain severity in chronic PHP: younger ages were associated with higher pain severity possibly due to pain perception. Previous studies found that chronic pain was more bothersome and distressing to younger adults than to the older age group [43,44]; however, no significant associations between age and pain severity for PHP were previously reported probably due to small sample size [18].

Quality of life was identified as another factor related to pain intensity and psychological health (i.e., level of depression), while foot function was also associated with age and psychological health. Similar to previous studies [33,45,46,47], a negative correlation between the quality of life and chronic PHP intensity was found. The deterioration in the quality of life, except in terms of mental health, previously reported [33], supported associations between psychological health and quality of life found in the network study. In addition, the fact that individuals with PHP may have perceived it as a mild injury without an important impact on specific aspects of mental health [33] could also explain these findings. Nevertheless, previous studies also found associations between foot function and depression in PHP [18,45], reporting that depression severity contributed 6% to the variance in function in PHP [18]. Thus, depression should be considered in preventive and treatment programs for PHP. However, it should be considered that depressive levels found in the present study, similar to those previously reported [18,45], were relatively small, explaining associations between psychological health and other variables reflected. Regarding age and foot function, positive correlations were found, as also previously reported [18]: older age was a demographic feature associated with foot function in PHP. In fact, it was revealed that age could contribute 8% to the variance in function in PHP [18], suggesting that rehabilitation programs should consider age in the proposal of treatment strategies to improve the management of PHP.

The data also showed that the number of TrPs was associated with quality of life and mechanosensitivity. Although this is the first study showing associations between the number of TrPs and mechanosensitivity (PPTs) in PHP, the relationship between both variables was previously reported in different musculoskeletal pain conditions, such as shoulder impingement [48] and lateral epicondylalgia [49]. Previous studies showed that a greater number of active TrPs was associated with pain intensity and related disability in PHP [12], suggesting an important role of TrPs in PHP. In fact, a greater number of muscle TrPs could presume the presence of spatial pain summation related to peripheral and central sensitization, as previously reported in other conditions [48], since TrPs could be considered as prolonged and relevant peripheral nociceptive inputs triggering sensitization mechanisms related to widespread pain and spatial pain summation [50,51]. Thus, improvements in PPTs related to TrPs treatment for PHP previously found [52,53,54] could confirm the role of TrPs in peripheral and central sensitization in PHP, supporting associations between TrPs and PPTs shown in this study. Additionally, previous results also showed improvements in the quality of life related to TrPs treatment [55,56], supporting the association between these variables and suggesting that this approach may be effective for the management of PHP.

The network identified that worst pain intensity showed the highest strength centrality followed by PPT at tibialis anterior and foot function, while PPT at tibialis anterior and sural nerve closely followed by worst pain intensity and depression showed the highest closeness centrality. Similar findings were found in betweenness centrality, where worst pain intensity and PPT at tibialis anterior were highlighted nodes. In this scenario, pain severity and mechanosensitivity seem to play a key role in PHP, so if clinicians want to influence other variables, e.g., those related to foot function also associated with psychological health, the best variables to focus treatment on would be pain severity and mechanosensitivity. In addition, mechanosensitivity could induce the fastest changes in other variables, e.g., those related to pain severity, foot function, and depression. In line with these findings, previous studies revealed that PPT was a significant predictor of pain severity or foot function in PHP, and depression was considered a predictor of foot function [18], suggesting the importance of a comprehensive therapeutic approach targeted on these variables in PHP. Thus, the management of PHP focused on these outcomes could improve current treatment strategies and promote an effective and comprehensive therapeutic approach in PHP.

### Limitations

Although this is the first study using network analysis in PHP, and despite the positive aspects of its use, some limitations should be recognized. First, the fact that this was a cross-sectional study precludes the ability to disentangle between–subjects from within–subjects relationships. However, network analysis could provide indicative potential causal pathways [19]. Second, although we included multiple aspects, such as pain-related, function, clinical, mechanosensitivity, psychological, and health-related variables, other variables (e.g., catastrophism), which could give a broader perspective of the biopsychosocial model approach in PHP, were not included. Third, we have only tested sensitivity to pressure pain, a static outcome of nociceptive gain. Thus, it would be interesting to investigate other dynamic outcomes related to sensitization, e.g., conditioning pain modulation or nociceptive flexor reflex, to determine different associations. Finally, we included the minimum participants per node for acceptable analyses. Larger sample sizes would result in more stable and accurate networks [57].

## 5. Conclusions

The application of network connectivity analysis in a sample of patients with PHP revealed a model where pain-related, function, clinical, mechanosensitivity, psychological, and health-related variables were internally associated with weak strength. The network showed that pain severity and mechanosensitivity were the nodes with the highest centrality measures, supporting a relevant role of pain and sensitization mechanisms in the model. These findings support that management of individuals with PHP should include comprehensive therapeutic approaches targeting all the aspects identified in the model.

## Figures and Tables

**Table 1:** Participants’ demographic and clinical characteristics values.

Variable	Sample	Data Distribution
Age (years)	Mean (SD): 42.2 (13.2) Median (IQR): 43 (20)	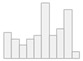
Gender (male/females)	Males: 41 (50.6%) Females: 40 (49.4%)	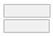
Pain Intensity after rest (0–10)	Mean (SD): 5.6 (1.9) Median (IQR): 6 (3)	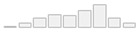
Pain Intensity after the first step (0–10)	Mean (SD): 6.2 (2.1) Median (IQR): 7 (3)	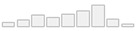
Worst Pain Intensity (0–10)	Mean (SD): 7.4 (1.9) Median (IQR): 8 (2)	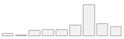
Foot Function Index (0–100)	Mean (SD): 40.7 (18.1) Median (IQR): 40.1 (22.5)	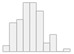
Beck Depression Inventory (0–63)	Mean (SD): 9.8 (9.4) Median (IQR): 6 (12)	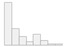
EuroQol-5D-5L (0–1)	Mean (SD): 0.7 (0.2) Median (IQR): 0.7 (0.1)	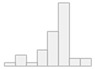
PPT Tibialis Anterior (kg/cm^2^)	Mean (SD): 3.1 (1.5) Median (IQR): 2.6 (1.9)	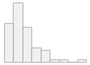
PPT Sural Nerve (kg/cm^2^)	Mean (SD): 2.0 (1.3) Median (IQR): 1.6 (0.9)	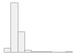
PPT Plantar Fascia (kg/cm^2^)	Mean (SD): 2.9 (1.1) Median (IQR): 2.8 (1.1)	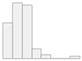
PPT Calcaneus Bone (kg/cm^2^)	Mean (SD): 4.0 (1.7) Median (IQR): 3.9 (1.9)	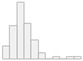
Trigger Points (n)	Mean (SD): 2.7 (2.8) Median (IQR): 2 (3)	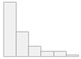

## Data Availability

All data derived from this study are presented in the text.
